# Effects of Normalizing Temperature on Microstructure and Impact Toughness of V-N Micro-Alloyed P460NL1 Steel

**DOI:** 10.3390/ma16216921

**Published:** 2023-10-28

**Authors:** Xinliang Li, Huibing Fan, Qiuming Wang, Qingfeng Wang

**Affiliations:** 1 State Key Laboratory of Metastable Materials Science and Technology, Yanshan University, Qinhuangdao 066004, China; 15805175186@163.com (X.L.); 18332576552@163.com (Q.W.); 2 Nanjing Iron & Steel Co., Ltd., Nanjing 211500, China; 15033576807@163.com; 3 National Engineering Research Center for Equipment and Technology of Cold Strip Rolling, Yanshan University, Qinhuangdao 066004, China; 4 Hebei Key Laboratory for Optimizing Metal Product Technology and Performance, Yanshan University, Qinhuangdao 066004, China

**Keywords:** normalizing temperature, P460NL1, microstructure, impact toughness

## Abstract

In this work, the influence of normalizing temperature on vanadium micro-alloyed P460NL1 steel is studied in terms of microstructures and impact toughness. With the normalizing temperature increased from 850 °C to 950 °C, the V(C,N) particles are dissolved. The dissolution of V(C,N) particles leads to a reduction in their ability to pin the primitive austenite grain boundaries, resulting in the coarsening of the primitive austenite grain. Simultaneously, the number of precipitated particles promoting ferrite nucleation decreased. The combination of these two effects led to the coarsening of ferrite grains in the steel samples. Of note, in the sample normalized at a temperature of 850 °C, the ferrite and pearlite crystals clearly exhibited banded structures. As the normalizing temperature increased, the ferrite–pearlite belt phase weakened. The highly distributed belt phase resulted in poor impact toughness of the steel sample normalized at 850 °C. The belt phase was improved at a normalizing temperature of 900 °C. In addition to that, the microstructure did not undergo significant coarsening at this normalizing temperature, thereby allowing it to achieve the highest toughness among all samples that were prepared for this study. The belt phase almost vanished at the normalizing temperature of 950 °C. However, microstructure coarsening occurred at this temperature, resulting in the deterioration of impact toughness.

## 1. Introduction

Rapid development of equipment and huge advances in thermo-mechanical control process (TMCP) technology have allowed the development of high-performance micro-alloyed steel via the hot-rolling process [1]. However, normalized steel plates with stabilized microstructures are still required in applications such as high-pressure vessels and tank trucks [2,3]. Hence, the strength improvement of normalized steel plates holds great significance, not only for improving building quality but also for reducing the self-weight of steel structures.

As a weldable, fine-grain steel normalized according to the designation EN10028-3 [4], P460NL1 (normalized steel) is one of the container steels that exhibits the highest strength levels reported so far. Indeed, a tank developed using P460NL1 has significantly reduced wall thickness in comparison to those developed using other types of steel. High-strength normalized steels with superior mechanical performance are urgently needed to meet ever-increasing demands. However, the delivery conditions of normalization impose some limitations on the strengthening methods, which means that controlled rolling and online accelerated cooling methods generally tend to be ineffective. Currently, strength improvement of normalized steel plates relies on alloying, among which V-N micro-alloying is an effective way to improve the strength of these materials [5,6,7,8,9]. The addition of N to steel can enhance the precipitation-strengthening effect of V and hence significantly increase the strength of steel [10,11]. During the heating process of steel, the undissolved V(C,N) particles prevent the grain from growing up [12,13]. Thereafter, the undissolved V(C,N) particles form a heterogeneous core of ferrite structures during the cooling process of normalization, inducing ferrite nucleation in the grains, thus promoting the refinement of ferrite grains [14,15]. This leads to an increase in the strength of the steel plate and thus improves its impact toughness. However, the normalizing process greatly affects the number, size, and distribution of the V(C,N) precipitated phases, significantly changing the microstructure and mechanical properties of the formed steel [6,16]. At high normalizing temperatures, the solubility of V(C,N) particles increases, and the number of V(C,N) particles in the steel decreases accordingly. This leads to a weakened ability of the particles to pin the primitive austenitic grain boundaries [17]. Meanwhile, the driving force in the growth of the primitive austenitic grains increases at elevated normalizing temperatures. Hence, an increase in normalizing temperature may cause severe coarsening of the normalized steel microstructure, resulting in deterioration of its impact toughness [8,18]. However, with the increase in normalizing temperature, the ferrite–pearlite band structure produced in the rolling process can be eliminated, and the impact toughness of the steel is improved [8,19]. Dong et al. [20] studied the effect of normalizing temperature on XG630DR. The results show that as the normalizing temperature increases, the impact energy of the test steel first increases and then decreases, and the impact toughness of the experimental steel is the best at 870 °C normalizing. Unfortunately, there are only a few reports so far that are devoted to a systematic study of the effect of normalizing temperature on the structure and the impact toughness of V-N micro-alloyed normalized steels.

Therefore, the effects of the normalizing temperature on the microstructure and mechanical performance of P460NL1 have been explored in detail in this study, providing references to the process optimization of P460NL1.

## 2. Experiment

### 2.1. Materials

To study the effect of normalizing temperature on the microstructure and the impact toughness of P460NL1, a 150 kg vacuum melting furnace was employed to smelt one furnace of a typical N content steel sample. The specific chemical compositions of this sample are given in Table 1. First, the smelted steel was cast into a rectangular ingot with dimensions of 220 mm (thickness) × 260 mm (width) × L mm (length of the ingot). After casting, the ingot was austenitized by heating it to a temperature of 1200 °C in a resistance furnace for a dwell time of 2 h. The ingot was then rolled into a 24 mm thick steel via a controlled rolling process using a 350 mm hot rolling machine, after which it was naturally allowed to cool down to room temperature.

### 2.2. Method

#### 2.2.1. Heat Treatment

**Figure 1:**
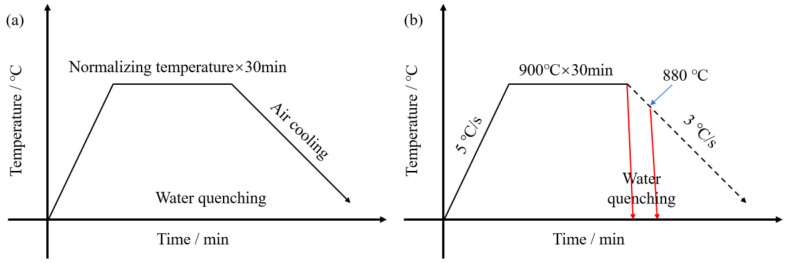
Schematic diagram of the normalizing process and simulated thermal cycle processes: normalizing process (**a**) and simulated thermal cycle processes on Gleeble 3800 (**b**).

Five plates with dimensions of 24 mm × 300 mm × 350 mm were cut from the rolled steel. The five plates were heated in a chamber resistance (YIANJIE ELECTRIC FURNACE INDUSTRY Co., Ltd., Luo Yang, China) for 30 min at temperatures of 850 °C, 875 °C, 900 °C, 925 °C, and 950 °C, respectively. The plates were then taken out and allowed to cool back to room temperature. A graphical figure depicting the specific process of normalizing heat treatment is provided in Figure 1a. Primitive austenitic grains of the samples at different normalizing temperatures, as well as phase transition, are observed to record changes in precipitation. The following thermal process is simulated on the Gleeble 3800 thermal simulator (Dynamic Systems Inc., New York, NY, USA). One pair of samples was heated to 900 °C at a speed of 5 °C/s, holding at 900 °C for 30 min. Then, the sample was quenched with water to 20 °C immediately. The other pair of samples was heated to 900 °C at a speed of 5 °C/s, holding at 900 °C for 30 min. Then, the sample was quenched with water to 20 °C as the sample cooled to 880 °C along the dotted line (Figure 1b).

#### 2.2.2. Characterization of Mechanical Properties and Fracture Morphology

**Figure 2:**
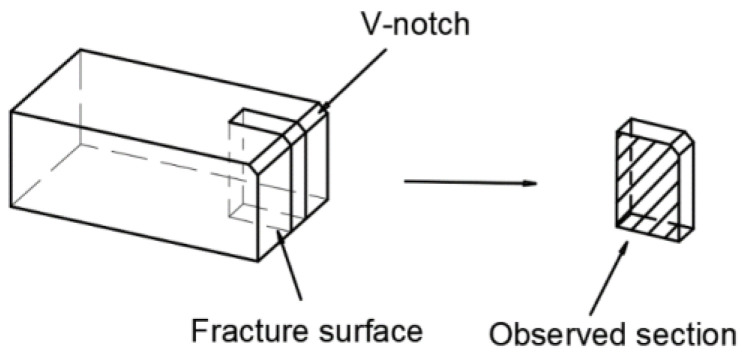
Schematic diagram of obtaining secondary crack sampling.

Three impact samples with dimensions of 10 mm × 10 mm × 55 mm were prepared along the transverse direction from five steel plates normalized at different temperatures. The impact energy of the samples at −40 °C was tested on a JBN-300B pendulum impact testing machine (DENGCE YIQI Co., Ltd., Jinan, China) according to the standard EN10028-3-2017 [4]. The average value was taken as the impact energy of the samples. The fracture morphology of the impact samples was then characterized using SU-5000 scanning electron microscopy (SEM, Hitachi Limited, Tokyo, Japan). Also, electron backscatter diffraction (EBSD, Hitachi Limited, Tokyo, Japan) was employed to study secondary cracks in the impact samples. The secondary crack sample was taken perpendicular to the fracture of the impact specimen, as shown in Figure 2. The sample was scanned using the EBSD system assembled on a scanning electron microscope with a 0.2 μm scan step size to characterize the secondary crack. (The EBSD sample preparation process is detailed in Section 2.2.3) A Vickers hardness tester (Jinan Heng Xu Testing Machine Technology Co., Ltd, Jinan, China) is used to determine the hardness of the plate at different locations along the thickness of the plate.

#### 2.2.3. Microstructure

Metallographic samples were prepared from the normalized steel plates of the prepared test steels at different normalizing temperatures. The samples were mechanically polished, etched with a 4% nitric acid alcohol solution for 5 s, and then placed under an optical microscope for observation. The metallographic samples were reground and electrolytically polished in an electrolytic solution, and then the electrolytically polished samples were scanned under a SU-5000 scanning electron microscope equipped with an EBSD with a 0.2 μm scan step size. The average grain size for each sample at different normalizing temperatures was calculated using the OIM software (Hitachi Limited, Tokyo, Japan). The microstructure of the samples that were quenched with water was observed via SEM. The main aim was to study the effect of normalizing temperature on phase transformation. The precipitated particles were extracted from the steel samples via the replica method and were then characterized using transmission electron microscopy (TEM, Japan Electronics optics Corporation, Tokyo, Japan) to investigate the effects of normalizing temperature on the precipitated particles. The preparation of the replica samples was carried out as follows: A thin and dense carbon film was sprayed on the surface of the metallographic specimen. The carbon film was split into a grid no larger than 3 mm × 3 mm. The sample containing the carbon film was immersed in an etchant (4% nitric acid alcohol solution) and etched to separate the carbon film from the sample. The carbon film was removed from the sample and fished out for transmission analysis [21].

## 3. Results

### 3.1. Effects of Normalizing Temperature on the Microstructure

**Figure 3:**
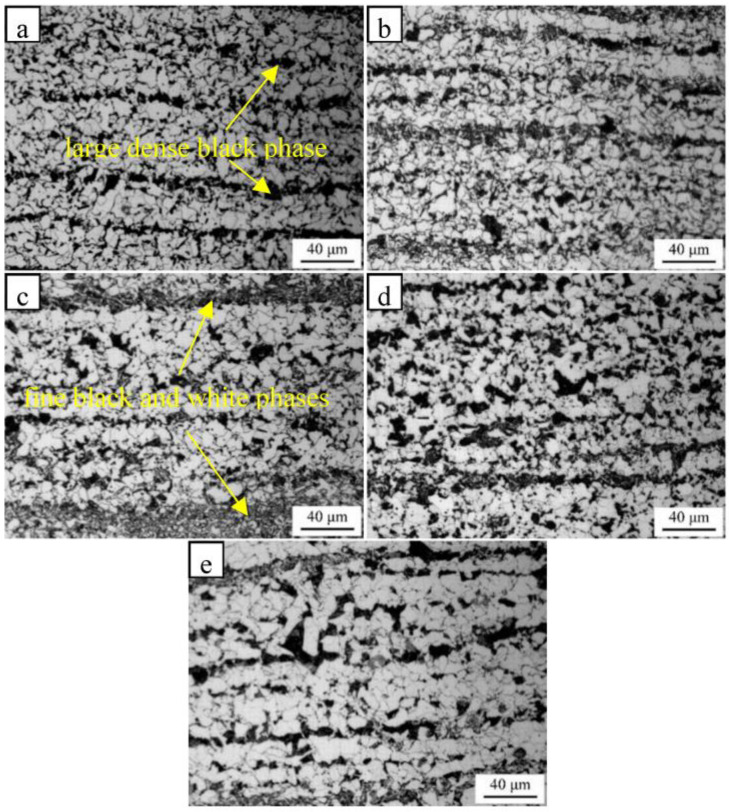
Microstructures of the samples normalized at different temperatures of (**a**) 850 °C, (**b**) 875 °C, (**c**) 900 °C, (**d**) 925 °C, and (**e**) 950 °C.

**Figure 4:**
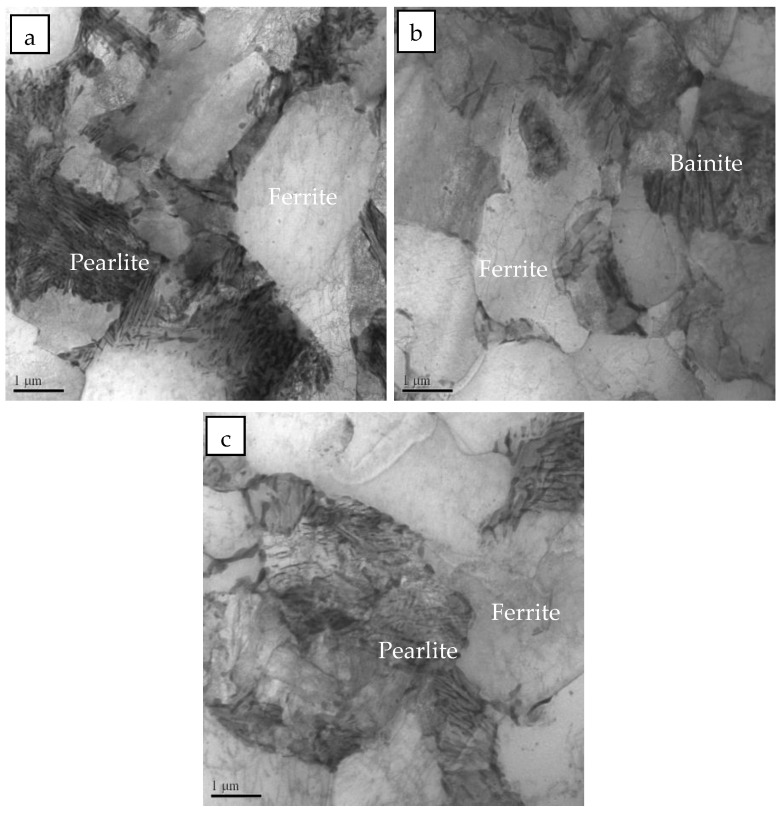
TEM of samples normalized at different temperature: (**a**) 850 °C, (**b**) 900 °C, and (**c**) 950 °C.

Images of the metallographic microstructure of the steel sample at different normalizing temperatures are provided in Figure 3. The microstructure in the specimens was further characterized using TEM, and the results are shown in Figure 4. The results show that the “large dense black phase” in the normalized samples is pearlite, and the “fine black and white phases” are bainitic ferrite. The microstructure of the sample heated at a normalizing temperature of 850 °C consisted of ferrites and pearlites. The samples heated at 875 °C and 900 °C were found to contain ferrite pearlites and bainitic ferrites, where the bainitic ferrite was banded and distributed. The pearlite content in the steel sample decreased, whereas the bainitic ferrite content increased as the normalizing temperature was increased from 850 °C to 900 °C. Consequently, grain refinement took place. As the normalizing temperature was further increased to 925 °C and 950 °C, the bainitic ferrite content in the sample gradually decreased while the pearlite content increased. The percentage composition for each microstructure was obtained using the Image-pro software (Image-Pro® Plus, Media Cybernetics, Bethesda, MD, USA). As the normalizing temperature increased from 850 °C to 900 °C, the pearlite content in the sample decreased from 17.2% to 3.2%, whereas the bainitic ferrite content increased from 1.7% to 15.5%. Furthermore, as the normalizing temperature was increased from 900 °C to 950 °C, the pearlite content in the test steel increased to 15.1%, while the bainitic ferrite content decreased from 15.5% to 5.6%.

**Figure 5:**
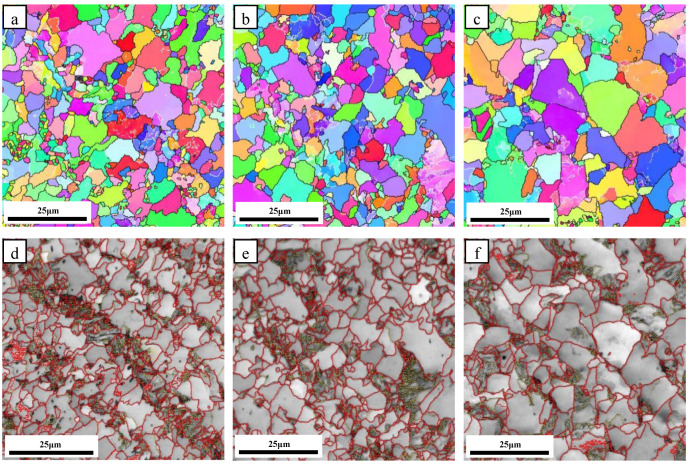
IPF and IQ images for the microstructures of steel samples normalized at different temperatures of (**a**,**d**) 850 °C, (**b**,**e**) 900 °C, and (**c**,**f**) 950 °C, (**a**–**c**) IPF, (**d**–**f**) IQ.

The inverse pole figures (IPF) and image quality (IQ) images with grain boundaries of the steel samples at different normalizing temperatures are provided in Figure 5. The red lines in Figure 5d–f represent the high-angle grain boundaries (HAGBs, misorientation angle ≥15°), whereas the yellow lines represent the low-angle grain boundaries (2~15°). As the normalizing temperature increased from 850 °C to 900 °C, grain coarsening was observed, leading to an increase in the average grain size from 5.7 μm to 6.6 μm. The areal density of HAGBs decreased from 0.49 μm^−1^ to 0.43 μm^−1^. An increase in the normalizing temperature from 900 °C to 950 °C led to severe grain coarsening, where the average grain size increased from 6.6 μm to 10.5 μm, and the areal density of HAGBs decreased from 0.43 μm^−1^ to 0.23 μm^−1^.

### 3.2. Effects of Normalizing Temperature on Mechanical Property

**Figure 6:**
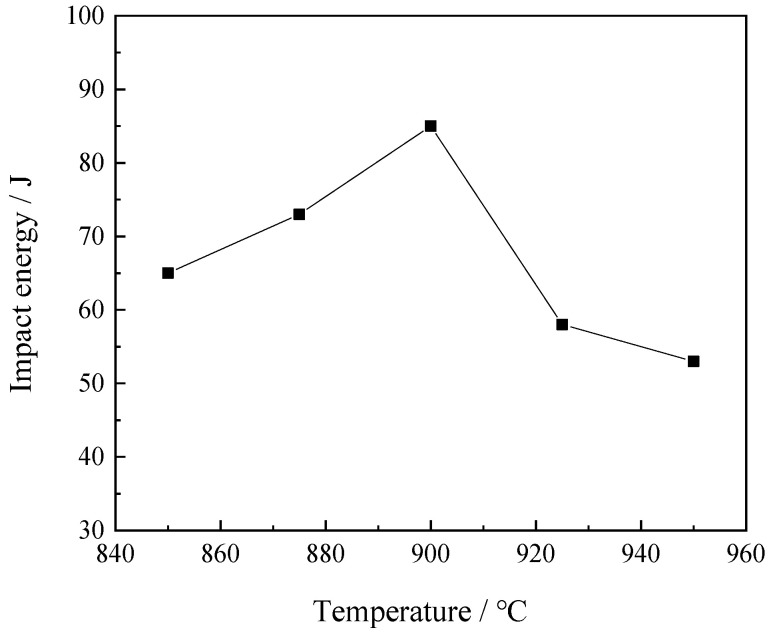
Impact energy of the steel samples as a function of the normalizing temperature.

Impact results for samples under −40 °C and normalized at different temperatures are summarized in Table 2 and Figure 6. The impact energies of samples normalized at temperatures of 850 °C, 875 °C, 900 °C, 925 °C, and 950 °C were found to be 65 J, 73 J, 85 J, 58 J, and 53 J, respectively. The impact energy of the steel samples initially increased almost linearly with an increase in normalizing temperature, achieving its maximum value at a temperature of 900 °C, after which it started to decrease.

**Figure 7:**
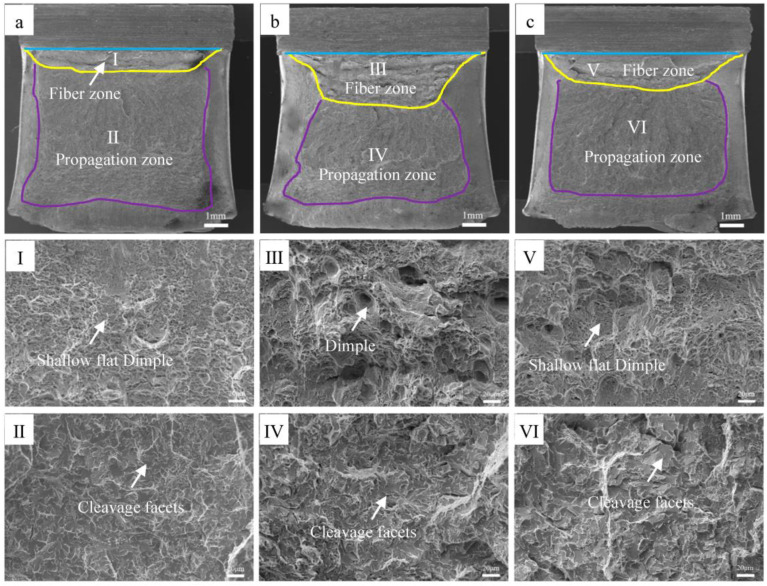
Impact fracture morphology of samples normalized at temperatures of (**a**) 850 °C, (**b**) 900 °C, and (**c**) 950 °C.

The fracture characteristics of the impact samples at different normalizing temperatures are shown in the SEM images presented in Figure 7. The impact fracture characteristics of the samples normalized at temperatures of 850 °C, 900 °C, and 950 °C are provided in Figure 7a, Figure 7b, and Figure 7c, respectively. Also, the area of the fracture fiber zone was computed using the Image-pro software. The fracture fiber rates for samples heated at normalizing temperatures of 850 °C, 900 °C, and 950 °C were obtained as 7%, 26%, and 15%, respectively. The fracture fiber of the impact samples increased initially and then decreased as the normalizing temperature was increased. The morphologies of the fracture fiber and the propagation zones shown in Figure 7a are depicted in Figure 7Ⅰ,Ⅱ, respectively. The fiber zone of the impact fracture at 850 °C was characterized by small and shallow dimples, while the propagation zone mainly consisted of large and flat cleavage planes. Similarly, the morphologies of the fracture fiber and the propagation zones in Figure 7b are shown in Figure 7Ⅲ,Ⅳ, respectively. The fiber zone of the impact fracture at 950 °C was also characterized by small and shallow dimples, whereas the propagation zone was mainly composed of cleavage planes. The morphologies of the fracture fiber and the propagation zones in Figure 7c are shown in Figure 7Ⅴ,Ⅵ, respectively. The fiber zone of the impact fracture for the sample heated at a normalizing temperature of 900 °C was characterized by small and deep dimples as well, while the propagation zone consisted of small cleavage planes. However, compared with the cleavage planes observed for the samples prepared at normalizing temperatures of 850 °C and 950 °C, the cleavage planes for the 900 °C sample were observed to be more “uneven”, and a tearing ridge with a few dimples was also found in between some of the cleavage planes.

## 4. Discussion

### 4.1. Effects of Normalizing Temperature on Microstructure

#### 4.1.1. Effects of the Normalizing Temperature on Precipitated Particles

**Figure 8:**
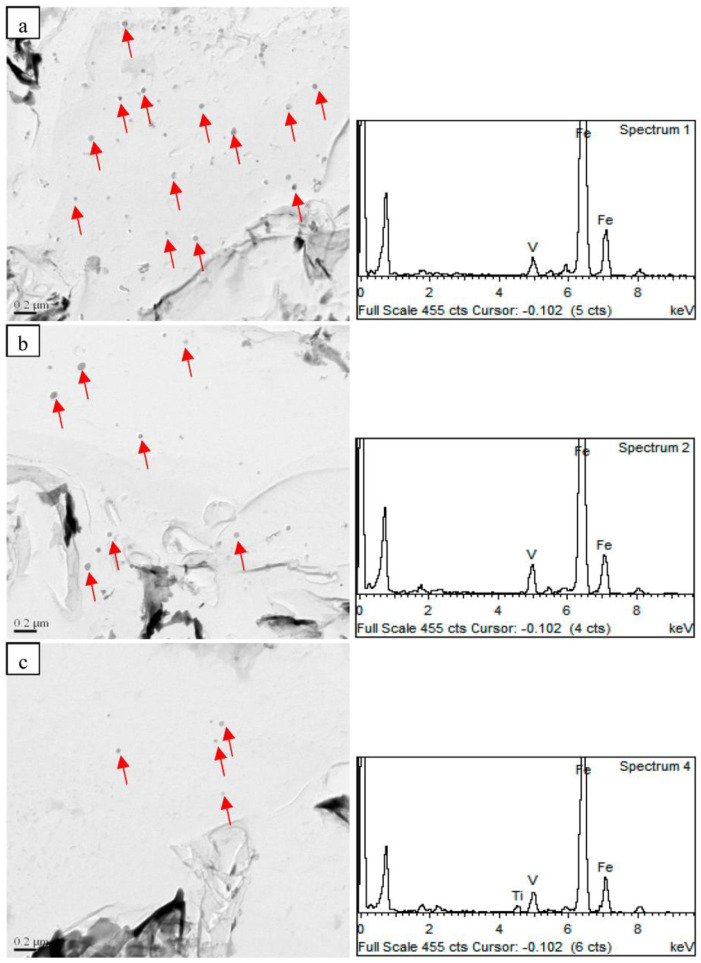
TEM images of precipitated particles normalized at different temperatures of (**a**) 850 °C, (**b**) 900 °C, and (**c**) 950 °C.

The obtained TEM images for precipitated particles in the samples normalized at different temperatures are provided in Figure 8. The precipitated particles in the steel samples were V(C,N) particles. The density of precipitated particles in the steel samples decreased from 21 μm^−2^ to 6 μm^−2^ as the normalizing temperature was increased from 850 °C to 950 °C. Meanwhile, the size of the precipitated particles decreased with an increase in the normalizing temperature. According to Formula (1) [22], the solubility of V(C,N) particles increased as the temperature was raised. This resulted in the dissolution of fine V(C,N) particles during the normalizing process, leading to a decrease in the number and size of precipitated particles in the steel samples.

(1) $$Log\left[V\right]\left[N\right]=3.46-\frac{8330}{T}$$

#### 4.1.2. Effects of Normalizing Temperature on Primitive Austenite Grains

**Figure 9:**
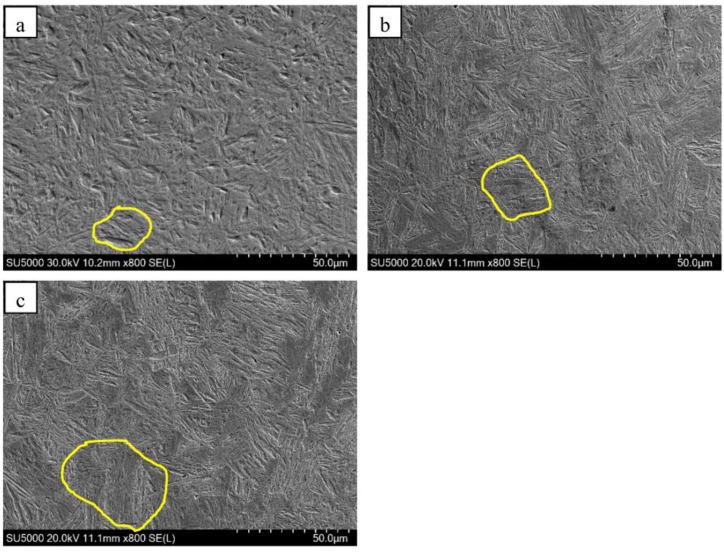
Primitive austenite grains of steel samples normalized at different temperatures of (**a**) 850 °C, (**b**) 900 °C, and (**c**) 950 °C.

Previous studies have shown that the grain size of normalized steel is closely related to the grain size of primitive austenite [17,23]. The microstructure of normalized steels can be improved significantly by refining the primitive austenitic grain size. So, in order to fully understand the effect of normalizing temperature on the microstructure of the test samples, it was necessary to first investigate its influence on the grain size of the primitive austenitic grains. The primitive austenitic grains of the steel samples after heating them at temperatures of 850 °C, 900 °C, and 950 °C for a dwell time of 30 min are shown in Figure 9. As the normalizing temperature increased from 850 °C to 900 °C, the grain size of primitive austenite increased from 21 μm to 32 μm. As the normalizing temperature further increased to 950 °C, the grain size of the primitive austenite was increased to 43 μm.

**Figure 10:**
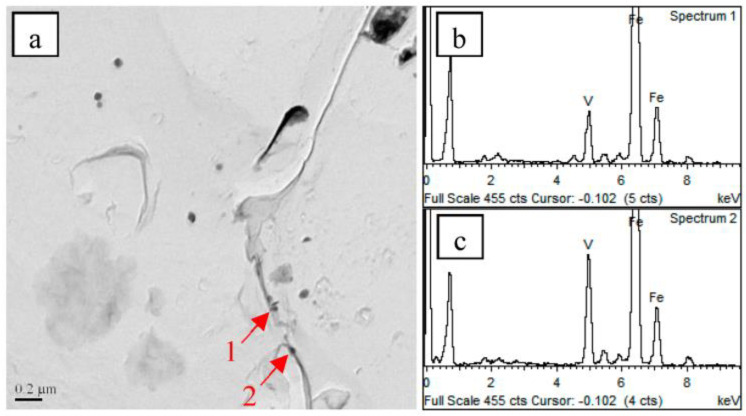
TEM micrographs showing precipitates (indicated by arrows) along grain boundary in carbon extraction replicas of the sample quenched with water at 900 °C (**a**), energy spectrum of particle 1 (**b**), and energy spectrum of particle 2 (**c**).

The coarsening of the primitive austenite grains depends on the dwell temperature of the steel samples and the dissolution of the precipitated particles. As shown in Figure 10, the finely precipitated particles can pinch the primitive austenite grain boundaries and inhibit the growth of the primitive austenite grains. However, as the normalizing temperature was raised, the precipitated particles in the steel samples dissolved. The number of particles thus decreased dramatically, leading to a reduction in their ability to pin the primitive austenite grain boundaries [24,25,26]. This lowered the resistance to the migration of the primitive austenite grain boundaries. Therefore, an increase in the normalizing temperature resulted in the coarsening of the primitive austenitic grains of the steel samples.

#### 4.1.3. Relationship of Primitive Austenite Grain Size with Precipitated Particles and Microstructure

**Figure 11:**
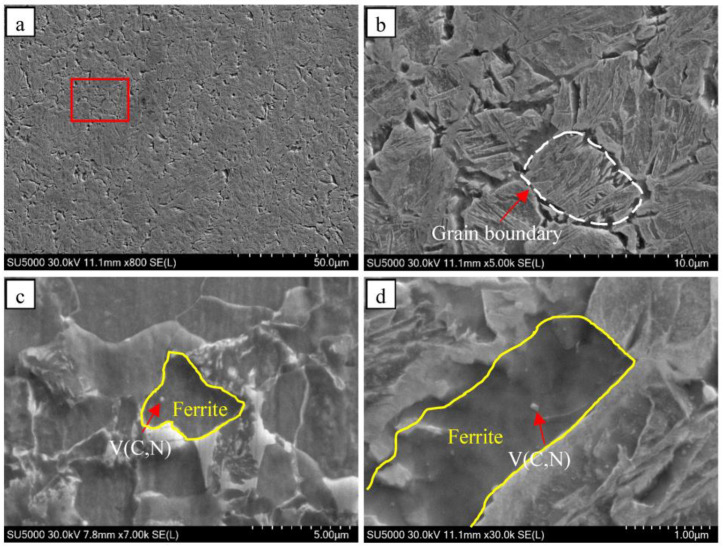
SEM images of sample quenched at 880 °C (**a**), enlarged in the red box in Figure 11a (**b**), intracrystalline ferrite nucleates on V(C,N) particle (**c**), and nucleation of grain boundary ferrite on V(C,N) particle (**d**).

The results of the microstructure morphology characterization of the thermal simulation samples after quenching at 880 °C are presented in Figure 11. According to Figure 11a, as the samples were cooled down to 880 °C, the ferrite transformation took place preferentially along the primitive austenite grain boundaries, which led to the formation of finer ferrite crystals. An enlarged view of Figure 11a of the area enclosed within the red box is provided in Figure 11b. It clearly shows that ferrite nucleation took place on the precipitated particles along the primitive austenite grain boundaries (Figure 11d). There are three main reasons why ferrite preferentially transforms at the primitive austenite grain boundary: (1) The energy at the primitive austenite grain boundary is higher than that of the matrix, which can provide more energy for ferrite nucleation to break through the nucleation barrier. (2) V(C,N) on the primitive austenite grain boundaries causes a further increase in energy at the grain boundaries. (3) The Baker Nutting orientation relationship between ferrite and V(C,N) ((001)_α_||(001)V(C,N), [110]_α_||[100]V(C,N)) reduces the interfacial energy [27]. Under the combined action of the above three aspects, the ferrite preferentially transforms on the precipitated particles at the primitive austenite grain boundaries. As shown in Figure 11c, the precipitated particles within the primitive austenite grains can also provide nucleation sites for the ferrite crystals and promote ferrite nucleation. The austenite grains in the steel samples grow in size with an increase in normalizing temperature, resulting in a decrease in density of the primitive austenite grain boundaries. The decrease in the density of primitive austenitic grain boundaries led to a reduction in the number of primitive austenitic grain boundaries that provided suitable locations for ferrite nucleation to take place. Meanwhile, the V(C,N) particles in the grains can also promote ferrite nucleation. As the normalizing temperature was raised, the V(C,N) particles in the steel samples were dissolved, and the number of precipitated particles promoting ferrite nucleation decreased as a result of the dissolution of the V(C,N) particles. The combination of these two effects led to the coarsening of ferrite grains in the steel samples as the normalizing temperature was raised.

### 4.2. Effects of Normalizing Temperature on Impact Toughness

**Figure 12:**
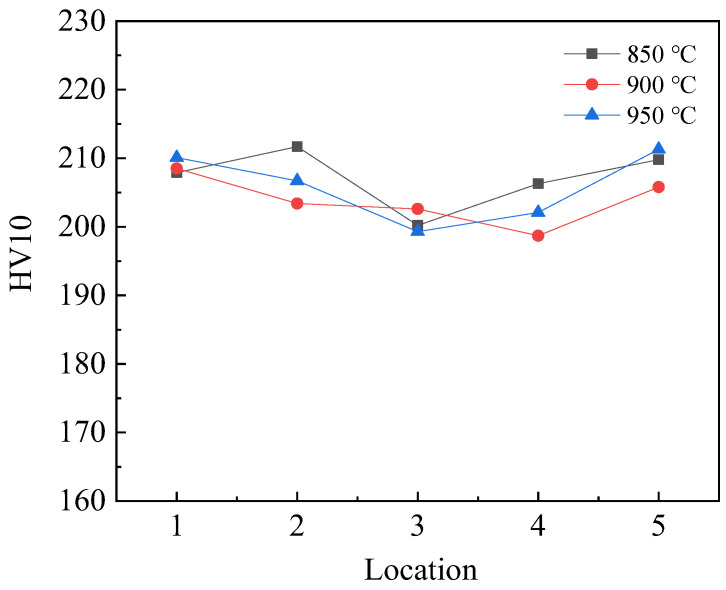
The hardness of steel plates with different normalizing temperatures along the thickness direction.

As the normalizing temperature increased from 850 °C to 900 °C, the impact energy of P460NL1 increased from 65 J to 88 J, and the fracture mode changed from brittle fracture to mixed-mode fracture. As the normalizing temperature was raised further, the impact energy of P460NL1 decreased from 88 J to 53 J, and the fracture behavior changed from mixed-mode fracture to brittle fracture. The non-uniformity of hardness along the thickness direction of the steel plate may be responsible for the difference in the impact toughness of the steel plate. Figure 12 shows the hardness test results of steel plates with different normalizing temperatures along the thickness direction. Figure 12, Figure 1, Figure 2, Figure 3, Figure 4, and Figure 5 represent the positions of the upper surface, upper 1/4, 1/2, lower 1/4, and lower surface of the steel plates along the thickness direction, respectively. There is almost no difference in the hardness of steel plates normalized at different temperatures, so the difference in hardness in the thickness direction is not the cause of the difference in the impact toughness of steel plates.

The impact energy can be divided into two parts: one is the crack initiation energy, and the other is known as the crack propagation energy. The effect of normalizing temperature on the impact properties of P460NL1 is discussed below from the point of view of crack initiation and propagation.

**Figure 13:**
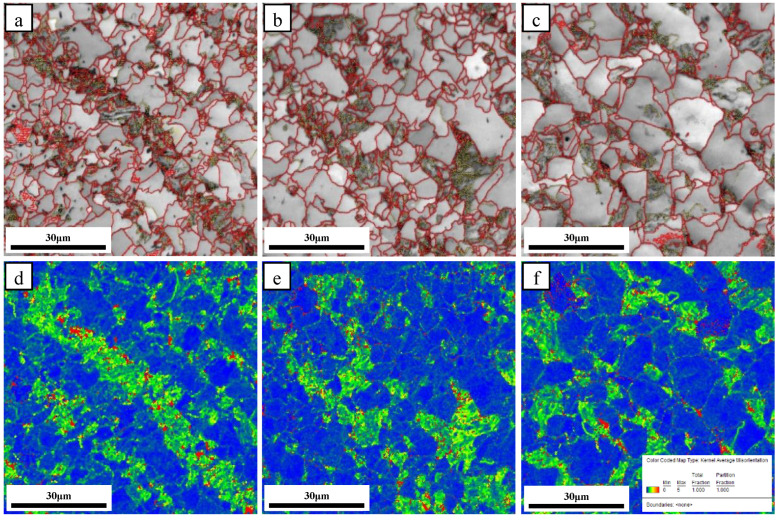
Microstructures and stress distributions for different samples at 850 °C (**a**,**d**), 900 °C (**b**,**e**), and 950 °C (**c**,**f**).

A great number of studies have shown that impact toughness is related to the second phase (M/A elements and pearlite), grain size, and microstructure composition [28,29]. The pearlite and ferrite shrink differently (different coefficients of thermal expansion) during the cooling process of normalizing. This led to a certain degree of strain within or around the pearlite structure, thus resulting in localized stress concentrations around or within the pearlite crystals [30]. The degree of strain within the pearlite crystals and in the matrix was measured using the kernel average misorientation (KAM) images, as shown in Figure 13.

It was observed that in the samples prepared at different normalizing temperatures, strain mainly built up at the pearlite or at the pearlite–ferrite interface. In the sample normalized at a temperature of 850 °C, the ferrite and pearlite crystals clearly exhibited banded structures, so that a continuous stress zone was formed in this sample. As the normalizing temperature increased to 900 °C, bainitic ferrite crystals started to form in the steel sample. The ferrite–pearlite belt phase was therefore weakened, and no continuous stress zone was formed here. The belt phase was further weakened as the normalizing temperature increased to 950 °C and the matrix stress became more uniformly distributed. Cracks were easily generated in the stress zone of the sample that was normalized at 850 °C, owing to the existence of a continuous stress zone [30]. Therefore, micro-crack initiation in these samples required lower energy in comparison to the samples prepared at normalizing temperatures of 900 °C and 950 °C.

**Figure 14:**
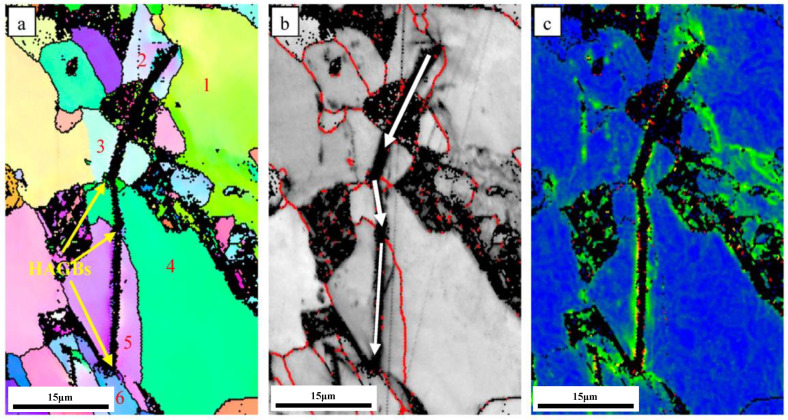
EBSD results for crack propagation in the samples prepared at a normalizing temperature of 900 °C. Inverse pole figures (**a**), band contrast map (**b**), and kernel average misorientation map (**c**).

The microstructure and spatial distribution of HAGBs play a significant role in hindering crack propagation [13,31]. The obtained EBSD images showing secondary cracks in the sample prepared at the normalizing temperature of 900 °C are provided in Figure 14, where the red line represents the high-angle grain boundaries (HAGBs) defined by an angle of 15°. The absence of deflection as the crack passes through the interior of the grains indicates that there is little obstruction to crack propagation inside the grains. Nevertheless, the direction of the crack propagation changed and was finally captured at HAGBs. The above results show that high-angle boundaries (θ > 15°) can act as effective barriers to impede crack propagation. In the case of the 900 °C sample, the ferrite grains were finer, the HAGBs were more closely spaced, and the cracks were deflected several times during the crack propagation. This can clearly be observed in Figure 14b (the white arrow represents the direction of crack propagation).

Due to the low yield strength of ferrite crystals, plastic deformation occurred during crack propagation as the ferrite structures absorbed the crack propagation energy and converted it into internal strain, thus consuming the crack propagation energy and hindering further propagation of the cracks (refer to Figure 14c). As the normalizing temperature was increased from 850 °C to 900 °C, the effective grain size of ferrite crystals in P460NL1 did not undergo significant coarsening, so that more energy would be consumed by cracks during the propagation process. As the normalizing temperature was raised to 950 °C, ferrite grain size increased drastically, while the areal density of HAGBs decreased. Hence, the crack propagation resistance was lower in the sample that was normalized at 950 °C, which led to lower energy consumption, thereby reducing its impact energy.

## 5. Conclusions

The conclusions are summarized as follows:

(1) The precipitated V(C,N) particles dissolved as the temperature was increased, resulting in the coarsening of primitive austenite grains and leading to a reduction in the content of ferrite nucleation particles. As a result, the ferrite grain sizes were coarsened.
(2) With the increase in normalizing temperature, the impact energy of the test steel first increases and then decreases, and the impact toughness of the test steel is at its best when the normalizing temperature is 900 °C.
(3) The severe banded structure of 850 °C normalizing steel results in poor impact toughness. The band structure of 900 °C normalized steel is improved, and the ferrite is not coarsened obviously, so the toughness of the test steel is the best. Although the banded structure of the 950 °C normalizing sample basically disappeared, the microstructure was obviously coarsened, which resulted in poor toughness of the test steel.
(4) Compared with ordinary micro-alloyed normalized steel, V-N micro-alloyed normalized steel has a finer grain size and better impact toughness.

## Figures and Tables

**Table 1:** Chemical composition of the prepared steel samples wt.%.

Steel	C	Si	Mn	P	S	Ni	V	N	Alt
165 N	0.183	0.26	1.70	0.009	0.002	0.51	0.162	0.0165	0.008

**Table 2:** Impact energy of steel samples normalized at different temperatures at −40 °C KV_2_/J.

Normalizing Temperature (°C)	850	875	900	925	950
Impact energy (J)	75 64 55/65	76 81 61/73	92 82 89/88	55 60 60/58	57 48 54/53

## Data Availability

The authors confirm that the data are available within the article.
